# Insight into Glycoside Hydrolases for Debranched Xylan Degradation from Extremely Thermophilic Bacterium *Caldicellulosiruptor lactoaceticus*

**DOI:** 10.1371/journal.pone.0106482

**Published:** 2014-09-03

**Authors:** Xiaojing Jia, Shuofu Mi, Jinzhi Wang, Weibo Qiao, Xiaowei Peng, Yejun Han

**Affiliations:** 1 National Key Laboratory of Biochemical Engineering, Institute of Process Engineering, Chinese Academy of Sciences, Beijing, China; 2 Institute of Agro-food Science and Technology, Chinese Academy of Agricultural Sciences, Beijing, China; 3 College of Biosciences and Biotechnology, Shenyang Agricultural University, Shenyang, China; Universidad de Granada, Spain

## Abstract

*Caldicellulosiruptor lactoaceticus* 6A, an anaerobic and extremely thermophilic bacterium, uses natural xylan as carbon source. The encoded genes of *C. lactoaceticus* 6A for glycoside hydrolase (GH) provide a platform for xylan degradation. The GH family 10 xylanase (Xyn10A) and GH67 α-glucuronidase (Agu67A) from *C. lactoaceticus* 6A were heterologously expressed, purified and characterized. Both Xyn10A and Agu67A are predicted as intracellular enzymes as no signal peptides identified. Xyn10A and Agu67A had molecular weight of 47.0 kDa and 80.0 kDa respectively as determined by SDS-PAGE, while both appeared as homodimer when analyzed by gel filtration. Xyn10A displayed the highest activity at 80°C and pH 6.5, as 75°C and pH 6.5 for Agu67A. Xyn10A had good stability at 75°C, 80°C, and pH 4.5–8.5, respectively, and was sensitive to various metal ions and reagents. Xyn10A possessed hydrolytic activity towards xylo-oligosaccharides (XOs) and beechwood xylan. At optimum conditions, the specific activity of Xyn10A was 44.6 IU/mg with beechwood xylan as substrate, and liberated branched XOs, xylobiose, and xylose. Agu67A was active on branched XOs with methyl-glucuronic acids (MeGlcA) sub-chains, and primarily generated XOs equivalents and MeGlcA. The specific activity of Agu67A was 1.3 IU/mg with aldobiouronic acid as substrate. The synergistic action of Xyn10A and Agu67A was observed with MeGlcA branched XOs and xylan as substrates, both backbone and branched chain of substrates were degraded, and liberated xylose, xylobiose, and MeGlcA. The synergism of Xyn10A and Agu67A provided not only a thermophilic method for natural xylan degradation, but also insight into the mechanisms for xylan utilization of *C. lactoaceticus*.

## Introduction

Xylan, the main hemicellulose component of plant cell wall, is a heteropolymeric polysaccharide consisted mostly of linear backbone of β-1,4-D-xylopyranoside units which are commonly decorated with 4-*O*-methyl-glucuronyl, acetyl, and arabinofuranosyl substituents [1], [2]. In a general sense, the efficient depolymerization of xylan to monosaccharides requires the synergistic function of enzyme system, including endo-β-1,4-xylanase (EC 3.2.1.8), β-xylosidase (EC 3.2.1.37), α-L-arabinofuranosidase (EC 3.2.1.55), α-glucuronidase (EC 3.2.1.139), and acetyl xylan esterase (EC 3.2.1.72) [1], [3]. Endo-β-1,4-xylanases catalyze the random cleavage of the internal β-1,4-glycosidic linkage between xylose residues in xylan polymer, and have been classified into glycoside hydrolase (GH) families 5, 7, 8, 10, 11 and 43 [4]. The α-glucuronidases (EC 3.2.1.139) cleave the α-1,2-linkage between 4-O-methylglucuronic acid (4-O- MeGlcA) and XOs [5]. Unlike xylanases, α-glucuronidases cluster into either GH family 67 or family 115 based on amino acid sequences [1], [6]. To date, a large number of xylanolytic enzymes have been identified from a variety of microbial sources (CAZy; http://www.cazy.org/). Thermostable hemicelluloytic enzymes, with a number of advantages over mesophilic enzymes, have thus gained worldwide industrial and biotechnological interest.

*Caldicellulosiruptor lactoaceticus* 6A, an anaerobic and extremely thermophilic, cellulose and hemicelluloses degrading bacterium, was isolated from an alkaline hot spring in Iceland [7]. It grows efficiently at temperature between 50 and 78°C and pH 5.8–8.2 within optimum near 68°C and 7.0, respectively. Besides xylan, it utilizes cellulose, starch, pectin, cellobiose, xylose, maltose and lactose as carbon sources. Thus its ability to express highly thermostable carbohydrate-active enzymes makes it an ideal candidate for studying extreme temperature biomass conversion. Currently, the complete genome sequence was available for this species [8], providing new approach to investigate the mechanisms of polysaccharides degradation. The genes encoding thermophilic GHs of *C. lactoaceticus* 6A provide a platform for degrading natural polysaccharides at higher temperature.

In present study, thermophilic degradation of MeGlcA decorated xylan using *C. lactoaceticus* 6A GHs was studied. The genes of endo-β-1,4-xylanase Xyn10A and α-glucuronidase Agu67A were identified and cloned in the genome of *C. lactoaceticus* 6A, and heterologously expressed, purified and biochemically characterized. The synergistically hydrolytic properties of the two enzymes on MeGlcA decorated xylan and XOs were also investigated.

## Results and Discussion

### Gene cloning and sequence analysis of Xyn10A and Agu67A

**Figure 1:**
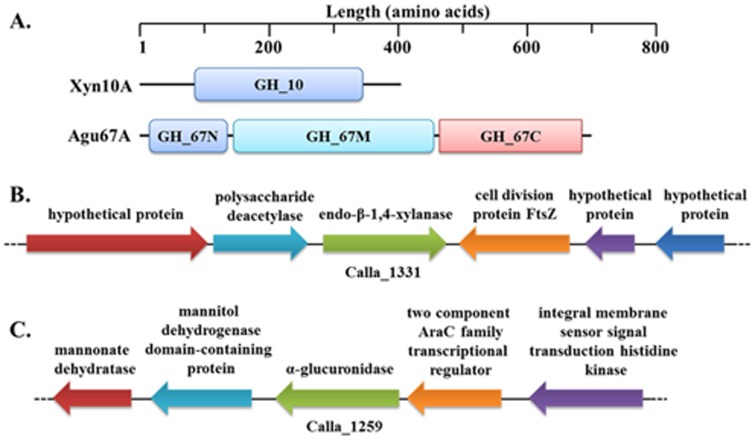
Modular and genomic organization of Xyn10A and Agu67A from *C. lactoaceticus*. A. Modular organization for Xyn10A and Agu67A. NCBI conserved domains database and SignalP 4.1 Server were used for analysis. B. Genomic organization for Xyn10A. Calla_1331 was annotated as a putative endo-β-1,4-xylanase, and upstream of the xylanase is the gene predicted to encode a polysaccharide deacetylase. C. Genomic organization for Agu67A. Calla_1259 was annotated as a putative α-glucuronidase. Gene annotations were performed using the rapid annotations using subsystems technology (RAST) Server.

**Figure 2:**
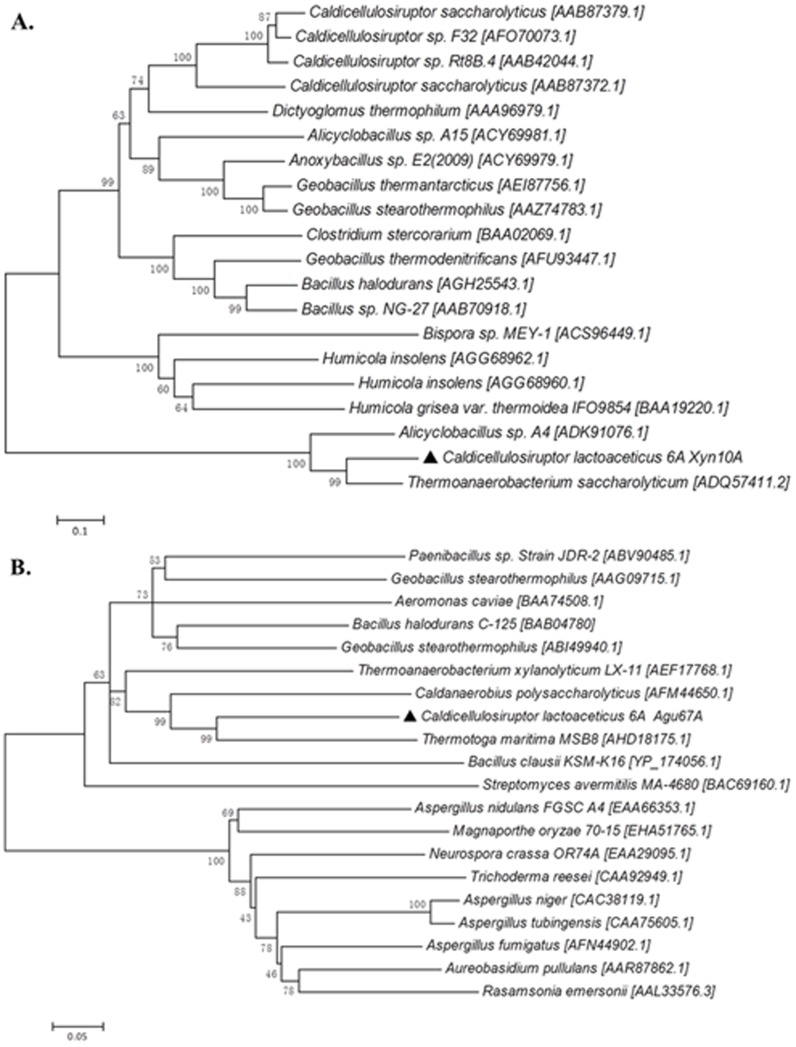
Phylogenetic analyses of Xyn10A and Agu67A. A. Phylogenetic tree of xylanases in different organisms. B. Phylogenetic tree of α-glucuronidases in different organisms. Trees were constructed using MEGA 5.05 by the Neighbor-Joining method with 1000 bootstrap replicates, and Genbank accession numbers of each protein sequence were given at the end of each species name.

Through *C. lactoaceticus* genome sequence analysis, Calla_1331 and Calla_1259 were annotated as putative GH10 endo-β-1,4-xylanase (Xyn10A) and α-glucuronidase (Agu67A), respectively. Both Xyn10A and Agu67A had no signal peptide, indicating they are intracellular enzymes. Xyn10A only contained a GH10 motif (Figure 1A), the calculated molecular weight (Mw) and deduced p*I* of Xyn10A were 46,965 Da and 5.65, respectively. The encoding gene *xyn*10A was amplified using *C. lactoaceticus* DNA as template. In genes screening analysis in genome DNA of *C. lactoaceticus*, no other xylan degradation genes except a putative polysaccharide deacetylase upstream of *xyn*10A was found (Figure 1B). In amino acids sequence blast analysis, Xyn10A showed high identity with other predicted xylanases from *Caldicellulosiruptor* sp. In addition to *Caldicellulosiruptor*, Xyn10A exhibited the highest similarity (79.7%) and identity (65.1%) with *Thermoanaerobacterium saccharolyticum* GH10 xylanase [GenBank: ADQ57411.2], and similarity (71.5%) and identity (57.8%) with GH10 xylanase from *Alicyclobacillus* sp. A4 [GenBank: ADK91076.1]. However, Xyn10A showed much lower similarity (27.0–41.8%) and identity (14.4–23.5%) with other characterized thermophilic GH10 xylanases, indicating that Xyn10A was a novel thermophilic GH10 xylanase. The amino acid residues Glu161 and Glu266, predicted acting as proton donor and catalytic nucleophile, were also conserved in Xyn10A [1], [9]. Phylogenetic analysis of defined thermophilic GH10 xylanases revealed the differentiation of the homology of thermophilic GH10 xylanases into three groups (Figure 2A). Xyn10A had the closest relationship with *T. saccharolyticum* xylanase, and then clustered with *Alicyclobacillus* sp. xylanase as a single evolutionary clade which was distinct from other thermophilic bacteria and fungi. On the contrary, Xyn10A was far isolated from other reported *Caldicellulosiruptor* sp. xylanase indicating the distinct interspecific-diversity of xylanases.

The C. *lactoaceticus agu*67A gene was predicted to encode a 693-amino-acid GH67 α-glucuronidase with Mw of 80,343 Da and a p*I* of 8.31. Agu67A had a conserved GH67 N-terminus (14–136 amino acids), a GH67 middle domain (146–467 amino acids), and a GH67 C-terminus (468–692 amino acids) motifs without signal peptide (Figure 1A). Genomic organization analysis did not find any xylanolytic genes around the *agu*67A gene (Figure 1C). Agu67A shared the highest similarity (79.9%) and identity (64.4%) to that of *Thermotoga maritima* MSB8 [GenBank: AHD18175.1], and had higher similarity and identity with other α-glucuronidase from *Caldanaerobius polysaccharolyticus* [GenBank: AFM44650.1] (77.1%, 61.4%), *Thermoanaerobacterium xylanolyticum* LX-11 [GenBank: AEF17768.1] (73.9%, 58.4%), *Geobacillus stearothermophilus* [GenBank: ABI49940.1] (73.2%, 58.2%), *Bacillus halodurans* C-125 [GenBank: BAB04780] (72.0%, 56.0%), and *Paenibacillus* sp. Strain JDR-2 [GenBank: ABV90485.1] (71.0%, 53.5%), respectively. Analysis of Neighbor-Joining (N-J) tree revealed Agu67A was located in the group of thermophilic bacteria (Figure 2B). Agu67A was closely related to *T. maritima* MSB8 α-glucuronidase, and then sub-clustered with *C. polysaccharolyticus* α-glucuronidase with reliable bootstrap values.

### Expression and purification of Xyn10A and Agu67A

**Figure 3:**
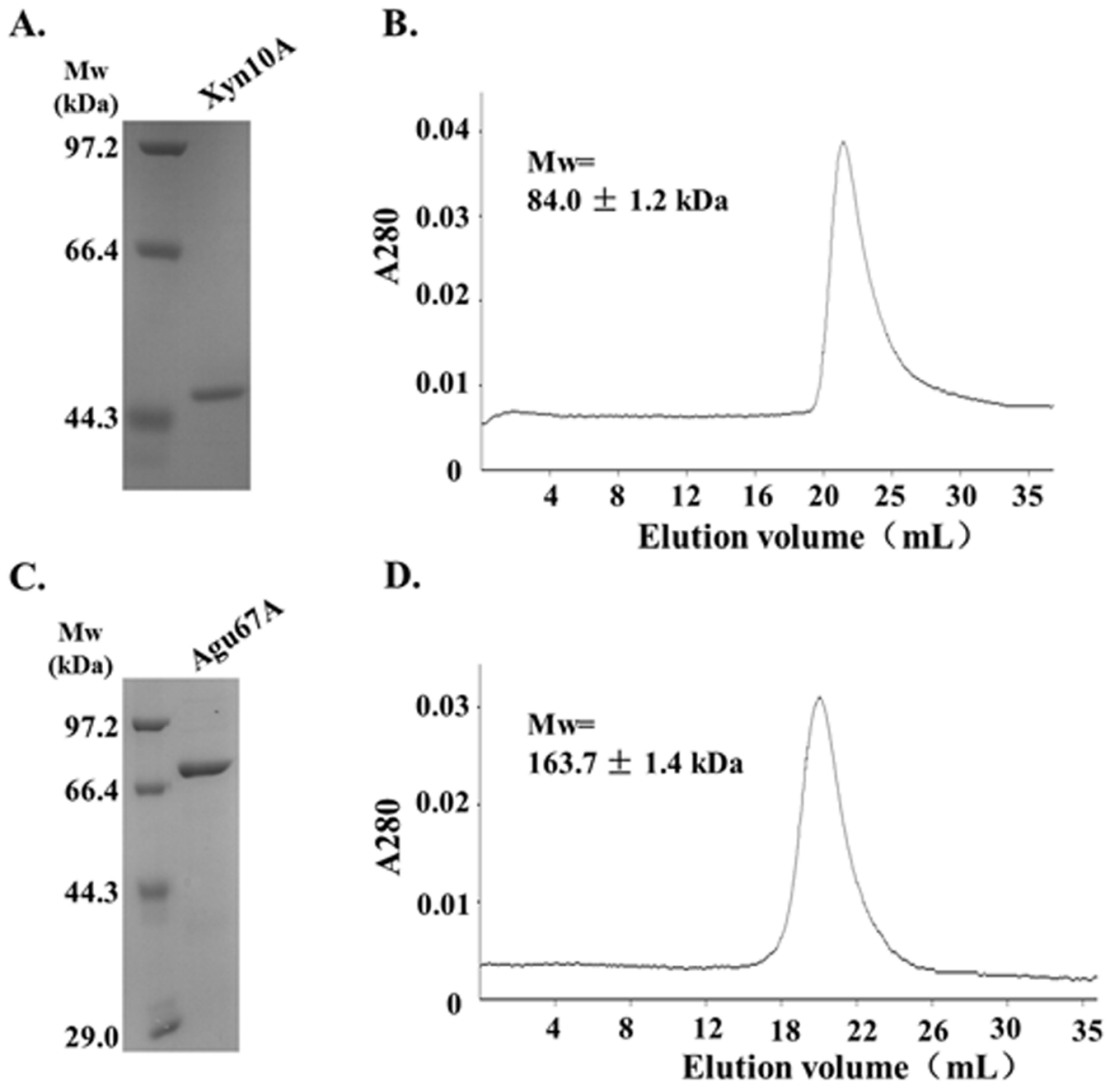
Purification of Xyn10A and Agu67A. A. SDS-PAGE analysis of purified Xyn10A. B. Quaternary structure analysis of Xyn10A by gel filtration chromatography. C. SDS-PAGE analysis of purified Agu67A. D. Quaternary structure analysis of Agu67A by gel filtration chromatography. Both Xyn10A and Agu67A were purified by Ni-affinity chromatography, followed by Superdex 200 gel filtration.

For functional analysis of the recombinant enzymes, both *xyn*10A and *agu*67A were sub-cloned into pET-28b vector and expressed in *Escherichia coli* BL21 (DE3). Proteins were heat-treated at 65°C for 30 min followed by Ni-affinity chromatography, and further purified through Superdex 200 gel filtration. The recombinant Xyn10A displayed as a single band with Mw of about 47.0 kDa by SDS-PAGE analysis, which was in agreement with the predicted Mw based on the amino acid sequence (Figure 3A). Size exclusion chromatography revealed that Xyn10A eluted as a single peak with Mw of 84.0±1.2 kDa, suggesting that Xyn10A existed as a homodimer in solution (Figure 3B). Other xylanases such as *Syncephalastrum racemosum* Cohn. (58.0 kDa) [10] and *Trichoderma reesei* (90.0 kDa) [11] are also homodimers, whereas the majorities are identified as monomeric proteins in solution, for instance, *Glaciecola mesophila* KMM 241 (43.0 kDa) [12], *Cohnella laeviribosi* HY-21 (42.0 kDa) [13], *B.halodurans* TSEV1 (40.0 kDa) [14], and *Remersonia thermophila* CBS 540.69 (42.0 kDa) [15].

SDS-PAGE analysis of the purified Agu67A indicated a single protein at approximately 80.0 kDa, which was also consistent with the theoretical Mw (Figure 3C). However, as shown in Figure 3D, Agu67A appeared as a single peak with Mw of 163.7±1.4 kDa, suggesting that Agu67A existed as a homodimer in solution (Figure 3D). It has already been proved that most bacterial α-glucuronidases, including *C. polysaccharolyticus* (158.0 kDa) [16], *Bacillus stearothermophilus* No. 236 (161.0 kDa) [17] and *B.stearothermophilus* T-6 (150.0 kDa) [18], consist of two subunits with Mw of around 75.0 kDa per subunit. In contrast, many fungal α-glucuronidases function as monomeric proteins with a higher Mw of 100.0 kDa per subunit due to the glycosylation [19].

### Substrate specificity of Xyn10A and Agu67A

**Figure 4:**
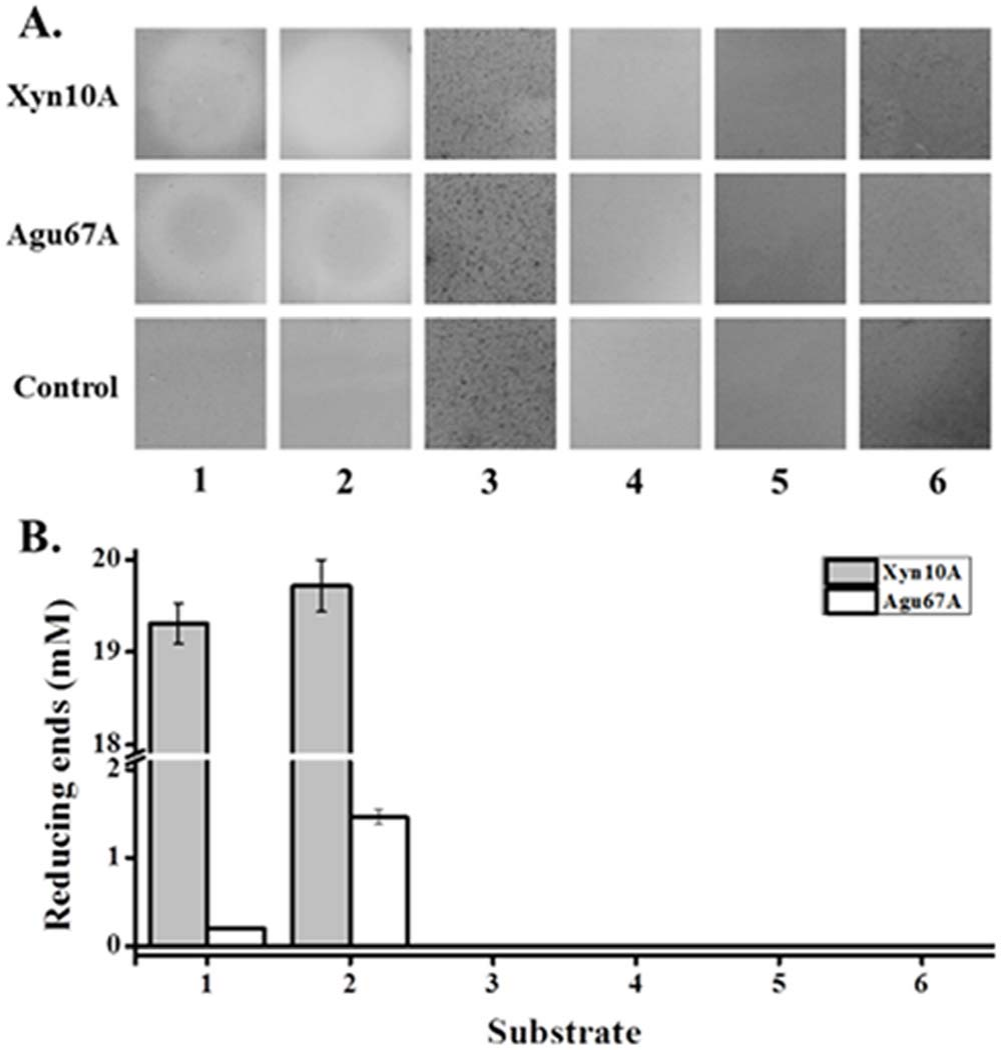
Hydrolytic activities of Xyn10A and Agu67A against different polysaccharide substrates. A. Identifications of Xyn10A and Agu67A activity on agar plate. The capacity of enzymes was assessed by incubating each protein on agar plates infused with different substrates at 60°C for 12 h, followed by staining with Congo red. B. The activity of Xyn10A and Agu67A on different substrates with produced reducing sugar assay. Both Xyn10A and Agu67A (0.5 µM each, final concentration) were incubated with different substrates at 80°C and pH 6.5 for 40 min. 1, beechwood xylan; 2, xylo-oligosaccharides; 3, locust bean gum; 4, soluble starch; 5, Avicel; 6, carboxymethyl cellulose. All of the tested substrates were at a fixed concentration of 1.0% (*w/v*).

Both Xyn10A and Agu67A were observed with clearing hydrolytic activity zones on the agar plates containing beechwood xylan or XOs (Figure 4A). In contrast, no clearing zones were found in the case of plates with LBG, soluble starch, Avicel, and CMC as substrates. Reducing sugars were detectable after incubation with beechwood xylan or XOs for 40 min with the Congo red assay (Figure 4B). Both Xyn10A and Agu67A displayed the highest activity with XOs as substrate. These results, therefore, suggested that Xyn10A and Agu67A possessed xylan degrading activity, but not mannanase, amylase, or cellulase activity.

It was reported that most of the α-glucuronidases are only active on MeGlcA linked short xylo-oligomers, while few studies have confirmed its high activity against polymeric substrates [20], [21]. The observable activity of Agu67A detected with beechwood xylan might mainly because of the existing of small amounts of aldobiouronic and aldotriouronic acids in the polysaccharide mixtures [16]. While the composition and structure of XOs depend on the xylan source and production process, enzymatic hydrolysis would produce branched hetero-xylooligosaccharides decorated with MeGlcA [22]. Typically, wood xylan exists as *O*-acetyl-4-*O*-methylglucuronoxylan in hardwoods with higher degree of polymerization (DP, 150–200), and as arabino-4-*O*-methylglucuronoxylan in softwoods [23].

### Biochemical characterization of Xyn10A and Agu67A

**Figure 5:**
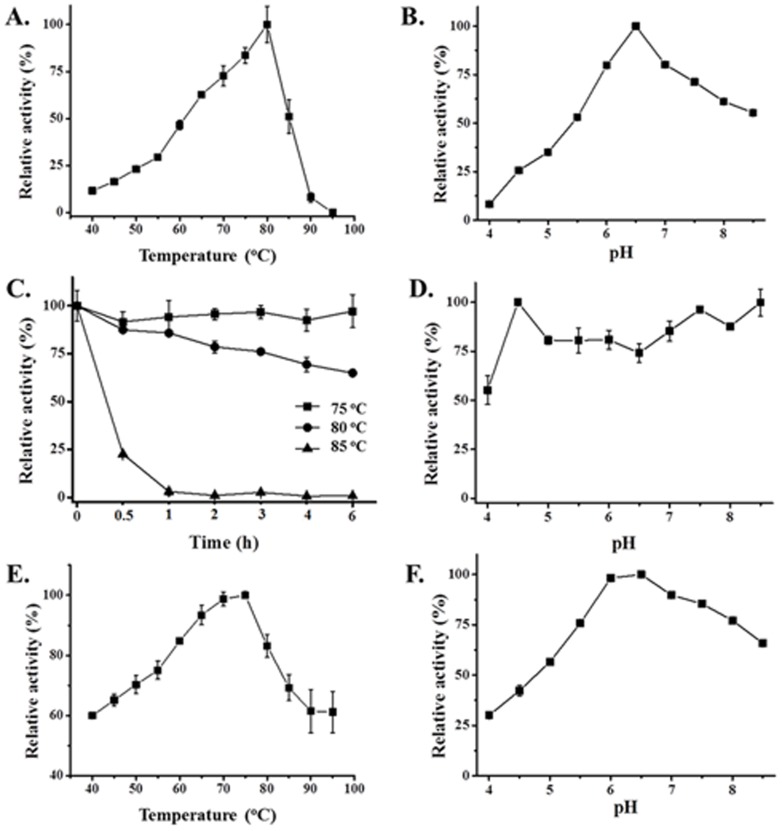
Effects of temperature and pH on the activity and stability of Xyn10A and Agu67A. A. Temperature profile of Xyn10A. Xylanase activity determination was performed in a temperature range of 40–95°C at pH 6.0 for 3 min. B. pH profile of Xyn10A. Xylanase activity assay was carried out by a 3 min incubation using phosphate-citrate buffers (pH 4.0–8.5) at 80°C. C. Thermostability profile of Xyn10A. The purified Xyn10A was incubated in pH 8.5 buffer at 75, 80 and 85°C, respectively for 0.5, 1, 2, 3, 4 and 6 h, and residual activity was detected under optimal conditions. D. pH stability profile of Xyn10A. The purified Xyn10A was pre-incubated in pH 4.0–8.5 buffers at room temperature for 10 h, and then the residual activity was measured under optimal conditions. E. Temperature profile of Agu67A. The α-glucuronidase activity determination was performed in a temperature range of 40-95°C at pH 6.5 for 5 min. F. pH profile of Agu67A. The α-glucuronidase activity assay was carried out by a 5 min incubation using phosphate-citrate buffers (pH 4.0–8.5) at 75°C. The maximum activity was defined as 100% and values shown were the means of three replicates.

Xyn10A displayed the highest activity at 80°C and maintained more than 50% of its activity at 65–85°C (Figure 5A). Moreover, Xyn10A was incubated at 75, 80 and 85°C in the absence of substrate for thermostability determination. Xyn10A was found to be extremely thermostable with over 90% residual activity after incubation at 75°C for 6 h, and still retained approximately 60% activity after incubation at 80°C for 6 h, while lost rapidly of its activity after incubated after incubation at 85°C for 30 min (Figure 5C). Interestingly, Xyn10A was most active at pH 6.5 and retained over 55% activity at pH 6.0–8.5, suggesting it's active in neutral and weak alkaline solution (Figure 5B). Furthermore, for pH stability assay, pure enzyme was pre-incubated in pH 4.0–8.5 buffers for 10 h without substrates at room temperature. Xyn10A exhibited good stability at pH 4.5–8.5, while 45% percent of the activity was reduced after incubation at pH 4.0 for 10 h (Figure 5D). The specific activity of Xyn10A was 44.6 IU/mg with beechwood xylan as substrate at optimum conditions.

Certain properties of Xyn10A were compared with some other thermophilic xylanases from bacteria and fungi as shown in Table 1. Xyn10A showed good catalytic activity over a broad temperature and pH range. The properties of Xyn10A indicated that enzyme activity remained more stable at temperature below 85°C and pH range 4.5–8.5.

**Table 1:** Properties comparison of *C. lactoaceticus* Xyn10A and other thermophilic xylanases.

Microorganism	Mw (kDa) a	Optimum temperature (°C)	Optimum pH	Specific activity (U mg^−1^)	References
*Caldicellulosiruptor lactoaceticus*	47.0	80	6.5	44.6 b	this study
*Syncephalastrum racemosum* Cohn	29.0	50	8.5	1,402.0 c	[10]
*Thermotoga thermarum*	131.0	95	7.0	145.8 b	[2]
*Actinomadura* sp. strain Cpt20	20.0	80	10.0	712.0±58.0 b	[24]
*Streptomyces* sp. CS428	37.0	80	7.0	926,103.0 b	[25]
*Streptomyces rameus* L2001	21.1	70	5.3	4326.0±97.0 b	[26]
*Streptomyces olivaceoviridis* E-86	1,200.0	60	6.0	332.5 b	[27]
*Thermoanaerobacterium saccharolyticum* NTOU1	50.0	63	6.4	78.0±4.4 b	[9]
*Marasmius* sp	40.0	90	6.0	336.0±22.0 b	[28]
*Paecilomyces themophila*	25.8	75–80	7.0	936.0 b	[29]
*Thermomyces lanuginosus* CBS 288.54	26.2	70–75	7.0–7.5	895.0±21.6 b	[30]
*Volvariella volvacea*	39.0	60	7.0	67.3±0.8 b	[31]
*Bispora* sp MEY-1	∼70.0	60	3.0	2,463.0 b	[32]
*Aspergillus niger*	33.0	60	5.0	3,200.0 b	[33]

a Mw, molecular weight by SDS-PAGE.

b Value for beechwood xylan.

c Value for birchwood xylan.

In addition, the influences of various additives including metal ions and reagents on Xyn10A activity were also investigated (Table 2). Most of ions except 1 mM Fe^3+^ and Zn^2+^ showed obviously effects on Xyn10A activity. It was strongly activated by 1 mM or 5 mM NH_4_^+^, Na^+^, K^+^, Mg^2+^, Ni^2+^; 1 mM Fe^2+^; and 5 mM Ca^2+^. Furthermore, the addition of 1 mM Ca^2+^ exhibited a moderate elevation to the activity. On the contrary, other concentrations of ions, including 1 mM or 5 mM Co^2+^, Mn^2+^, Cu^2+^, and 5 mM Fe^2+^, Fe^3+^, Zn^2+^, significantly inhibited the xylanase activity. Reductant, detergents, and metal chelator also had influence on the enzyme activity. 1 mM or 5 mM DTT, 0.1% or 0.5% Triton X-100, 5 mM EDTA, along with 0.1% β-ME markedly as well as 1 mM EDTA slightly increased the activity. However, the activity was strongly interrupted by 0.1% or 0.5% SDS and 0.5% β-ME. In addition, both 5% and 10% glycerol showed the highest positive impact on xylanase catalytic activity. But it was noteworthy that the activity was almost completely inhibited by three tested organic reagents (ethanol, isopropanol, and butanol).

**Table 2:** Effect of various metal ions and reagents on the activity of Xyn10A.

Ions and Reagents	Concentration	
	1 mM	5 mM
Control a	100.0±0.9	100.0±0.9
NH_4_Cl	157.1±1.1	139.2±6.3
NaCl	125.8±7.9	148.7±8.0
KCl	124.9±4.4	150.2±7.7
MgCl_2_	122.4±6.8	166.3±2.5
FeCl_2_	119.1±0.3	27.8±1.0
CaCl_2_	111.9±8.2	130.0±7.2
FeCl_3_	106.2±5.8	90.6±4.0
ZnCl_2_	104.8±6.9	29.9±0.4
CoCl_2_	61.8±1.2	68.7±1.5
MnCl_2_	81.8±0.8	55.1±1.6
CuCl_2_	46.8±1.3	10.8±2.3
NiCl_2_	121.3±4.9	114.7±1.4
DTT b	142.3±5.1	141.7±0.4
EDTA b	113.8±4.5	107.7±2.0
	0.1% (v/v)	0.5% (v/v)
β-ME b	122.0±0.7	48.2±1.1
Triton X-100	159.6±2.5	136.5±3.4
SDS b	11.1±2.3	0.2±0.1
	5% (v/v)	10% (v/v)
Glycerol	187.0±3.5	193.9±2.8
Ethanol	14.9±1.9	0.3±0.1
Isopropanol	36.6±0.4	22.0±2.4
Butanol	4.5±0.9	0.04±1.05•10^−3^

a 100% was considered for the activity of recombinant Xyn10A without additives.

b EDTA: ethylenediamine tetraacetic acid; SDS: sodium dodecyl sulfate; β-ME: β-mercaptoethanol; DTT: dithiothreitol.

In the previous reports, a large number of xylanases were also affected to some extent by these metals which suggested its possible function as a cofactor for maintaining structure stability and aiding enzyme-substrate reaction [2]. Crystal structure of *Bacillus* sp. NG-27 extracellular GH10 endo-xylanase revealed a metal binding site located at the C-terminal end of the catalytic domain [34]. An Mg^2+^-binding site was seen and the xylanase activity had a concentration-dependent manner with the presence of Mg^2+^. Some metals, such as Cu^2+^ and Zn^2+^ had strong affinities toward sulphydryl groups hence strongly inhibited the activity [15]. Notably, Xyn10A activity was enhanced by disulfide-reducing agents (0.1% β-ME, 1 mM and 5 mM DTT), indicating that the presence of the thiol group was essential but not absolutely critical for its activity although 0.5% β-ME played an inverse role. Owing to the strong protein denaturation, little xylanases had high SDS resistance even at low concentration of SDS [31]. In the study of *Thermotoga thermarum* xylanase, the enzyme activity was found to be greatly stimulated by Ca^2+^, Mn^2+^ and Co^2+^ [2]. The *T. saccharolyticum* NTOU1 XynFCB activity was also enhanced by the metal ions NH_4_^+^, K^+^, Na^+^, and Ca^2+^, while strongly inhibited by Cu^2+^ and Zn^2+^ [9]. The presence of 1 mM Mn^2+^, β-ME and EDTA enhanced the *Alicyclobacillus* sp. A4 XynA4-2 activity, whereas 1 mM Zn^2+^, Cu^2+^, and SDS resulted in severe or complete inhibition [35]. Likewise, the activity of *Actinomadura* sp. strain Cpt20 xylanase was also found to be enhanced by Ca^2+^ and Co^2+^, but was inhibited by Fe^2+^, Zn^2+^, Cu^2+^ and Mg^2+^, yet almost unaffected by K^+^, Na^+^ and Mn^2+^ [24]. In contrast to the results obtained, ethanol, isopropanol and butanol had no effect on the *Streptomyces rameus* L2001 xylanase activity [26].

Optimal temperature and pH for Agu67A were 75°C (Figure 5E) and 6.5 (Figure 5F), respectively. The specific activity of Agu67A was 1.3 IU/mg with XOs containing aldobiouronic acid as substrate by analyzing the released xylose under optimal conditions. The *C.polysaccharolyticus* Agu67A was most active at 60°C and pH 5.5 with specific activity reaching 10.8 IU/mg and 11.6 IU/mg for (4-*O*-methyl-α-D-glucurono)-D-xylan and birchwood xylan, respectively [16]. Similarly, the temperature and pH profiles of *B. stearothermophilus* T-6 α-glucuronidase were 65°C and pH 5.5–6.0, respectively [18].

### Hydrolysis of beechwood xylan and XOs by Xyn10A and Agu67A

**Figure 6:**
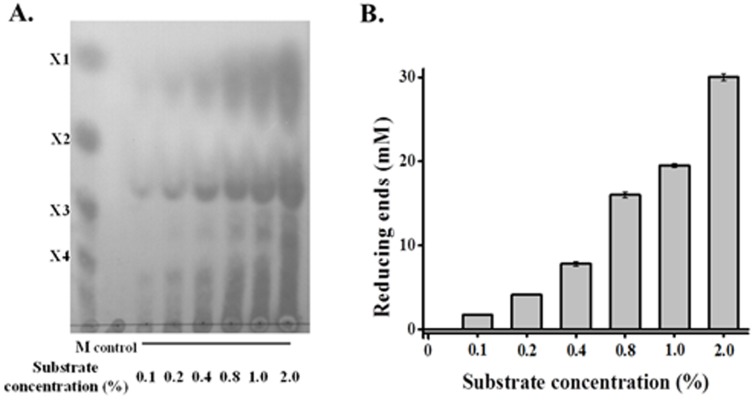
Hydrolysis of beechwood xylan at different concentration with constant loading of Xyn10A. A. TLC analysis of each hydrolysis products. B. The produced reducing sugar assay in each hydrolysis products. 0.1–2.0% (*w/v*) beechwood xylan was incubated with Xyn10A (0.5 µM, final concentration) at 80°C and pH 6.5 for 4 hours.

In order to assay the relationship between Xyn10A capacity and substrate abundance, different concentrations of beechwood xylan were hydrolyzed by constant amount of enzyme. Xylose, xylobiose, xylotriose, xylotetraose, as well as higher polymeric XOs, accumulated with the increasing of substrates (Figure 6A). Meanwhile, reducing ends also elevated quickly with more substrates addition, the results were also found in TLC analysis (Figure 6B).

**Figure 7:**
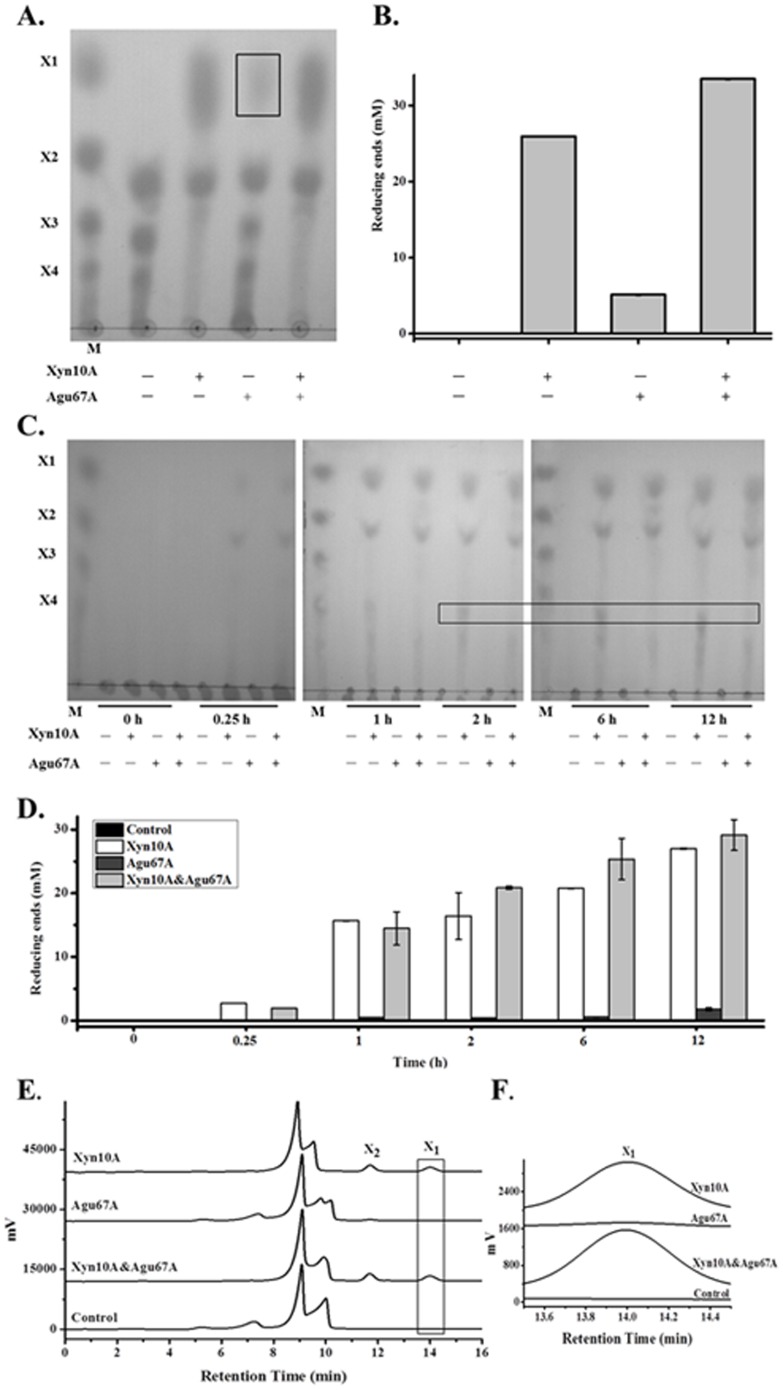
Hydrolysis products released from XOs and beechwood xylan by Xyn10A and Agu67A. A. TLC analysis of the XOs hydrolysis products. The detectable xylose produced by Agu67A was marked with a box. B. Produced reducing sugar assay of the XOs hydrolysis products. C. TLC analysis of the beechwood xylan hydrolysis products after different incubation times. D. Produced reducing sugar assay of the beechwood xylan hydrolysis products. The differences of hydrolysis products after 1 hour were marked with a box. E. HPLC analysis of the beechwood xylan hydrolysis products of 2 hours. F. Details of the HPLC analysis (retention time 13.5–14.5 min). XOs hydrolysis was performed by incubating single or mixed enzyme (2.0 µM each, final concentration) with 2.0% (*w/v*) XOs at 80°C and pH 6.5 for 4 hours. Beechwood xylan hydrolysis was conducted by incubating Xyn10A (1.75 µM, final concentration), or Agu67A (0.85 µM, final concentration), or Xyn10A (1.75 µM, final concentration) and Agu67A (0.85 µM, final concentration) mixture with 1.0% (*w/v*) beechwood xylan at 80°C and pH 6.5 for different times (0, 0.25, 1, 2, 6, 12 hours). Xylose (X_1_), xylobiose (X_2_), xylotriose (X_3_), and xylotetraose (X_4_) were used as standards and labeled.

Moreover, to explore their effects on XOs hydrolysis, the two enzymes were incubated separately or combination with XOs, and the products were analyzed. TLC analysis revealed the efficient break down of XOs into xylose and xylobiose in the presence of Xyn10A or two combined enzymes (Figure 7A). As aldobiouronic acid existed in XOs, a small amount of xylose was visible when XOs incubated with Agu67A. As a result, the release of reducing ends by Xyn10A, Agu67A, and Xyn10A&Agu67A were 26.0 mM, 5.1 mM, and 33.5 mM, respectively (Figure 7B). The synergistic activity of Xyn10A&Agu67A was also verified by results of HPLC analysis (*data not shown*). Xyn10A primarily degraded XOs into xylobiose and xylose, while Agu67A specifically digested aldobiouronic acid to MeGlcA and xylose. As a result, a slightly synergistic effect of xylose and xylobiose equivalents was seen when they coordinately acted together.

Furthermore, to evaluate the synergistic activity of Xyn10A and Agu67A, single or mixed enzymes were incubated with beechwood xylan and the products of hydrolysis were analyzed accordingly. As shown by the TLC analysis (Figure 7C), Xyn10A mainly produced xylose, xylobiose, a spot of heterogeneous XOs, and short chain polymers at the beginning. However, the products of Agu67A hydrolysis were undetectable throughout 12 hours incubation. Similarly, the synergistic activity of Xyn10A and Agu67A was detected in the process. When Xyn10A and Agu67A were applied together, the major hydrolysis products were xylose and xylobiose. The reducing sugar increased along the twelve hours reactions, and XOs disappeared after 1 hour reaction. The final reducing sugars were 27.0 mM, 1.8 mM, and 29.1 mM when Xyn10A, Agu67A, and Xyn10A&Agu67A were applied respectively (Figure 7D).

In addition, to further evaluate the synergistic activity of Xyn10A and Agu67A, the products of beechwood xylan hydrolyzed for 2 h were determined by HPLC (Figure 7E). Peaks representing xylose and xylobiose were observed when Xyn10A was applied alone, while the production by Agu67A was negligible (Figure 7F). In the reaction of Xyn10A&Agu67A applied together, the produced xylose and xylobiose was increased compared with that of enzyme added separately, indicating the synergism of the two enzymes. All these results clearly showed that Xyn10A acted on both xylan polymer and XOs, and liberate a large number of xylose and xylobiose, indicating Xyn10A was active on XOs with DP ≥3. While Agu67A was mostly active on branched XOs with methyl-glucuronic acids sub-chains, and primarily generated XOs equivalents. As had been stated, the synergism of Xyn10A and Agu67A would improve the end products of xylobiose and xylose taking MeGlcA branched XOs or xylan as substrates. While due to the complex structure of beechwood xylan, its hydrolysis was even more complex than that of MeGlcA decorated XOs.

Many GH10 xylanases were detected to degrade xylan polymer into a mixture mostly of xylose and XOs with DP lower than five [12]. The action mode of Xyn10A was in accordance with that of xylanase from *T. thermarum*, in which xylose and xylobiose were the major end products of beechwood xylan after 5 h hydrolysis [2]. Similarly, the hydrolysis products of birchwood xylan by *Alicyclobacillus* sp. A4 XynA4-2 contained 92.7% xylose and 7.3% xylobiose [35]. However, xylotriose and xylotetraose instead of xylose were formed largely from the hydrolytic reaction of birchwood xylan by *C. laeviribosi* HY-21 iXylC [13]. Xylobiose, along with higher XOs as intermediates, clearly detected as the predominant end products released by *Streptomyces* sp. CS428 Xyn428 from beechwood xylan [25].

Given the possibility that endoxylanases might breakdown xylan into XOs substituted with MeGlcA, microorganisms form α-glucuronidase to remove these side chains hence ensuring the effectiveness of xylan degradation. Nevertheless, a majority of the α-glucuronidases only has the capacity to act on small model xylan or MeGlcA branched XOs [17], [20]. Thus, the more reducing sugar detected in enzyme-cocktail treatment was related to the synergistic action of Xyn10A and Agu67A on the different parts of xylan polymer or XOs. The backbone-hydrolyzing Xyn10A liberates substituted XOs which are then degraded into monosaccharides by Agu67A and other xylanolytic enzymes. Synergism between xylanase and α-glucuronidase in hydrolysis of xylan has been investigated to some extent. In *C. polysaccharolyticus*, the release of either xylose or xylobiose tended to be slightly higher from birchwood xylan when incubated with two enzyme mixture than with single xylanase at 65°C [16]. The purified α-glucuronidase of *Aspergillus tubingensis* observed to liberate minor amounts of MeGlcA from birchwood xylan, and the amounts of MeGlcA and short oligo-saccharides (xylobiose and xylotriose) were improved in the two enzymes combination system [19]. The *Schizophyllum commune* α-glucuronidase was able to remove the MeGlcA groups from polymeric glucuronoxylan, and 76% of MeGlcA side groups were cleaved from the backbone of glucuronoxylan together with a xylanase [20].

*C. lactoaceticus* 6A is an extremely thermophlic plant biomass-degrading bacterium, with capability of utilizing xylan as carbon source [7]. Analysis of the genome of *C. lactoaceticus* revealed only three genes encoding endo-β-1,4-xylanase (Calla_1331, Calla_1781, and Calla_0206). Both Calla_1781 and Calla_0206 contained one GH10 domain and two to three carbohydrate-binding modules (CBMs). Interestingly, all of the three xylanases were predicted to be intracellular based on the absence of signal peptides. Specifically, no genes encoding putative β-xylosidase were annotated throughout the genome. Hence, other xylan-specific enzymes, as well as their novel synergistic roles need to be further investigated to give a deeper understanding of the mechanisms involved in xylan deconstruction process. In previous study, Calla_1781 was also expressed in *E. coli* BL21 (DE3) whereas no activity was detected (*data not shown*). Consequently, Calla_1331 (namely Xyn10A), the first characterized xylanase without CBMs, might played a prominent role in efficient degradation of xylan.

## Conclusions

In this study, two novel thermostable xylanolytic enzymes endo-β-1,4-xylanase Xyn10A and α-glucuronidase Agu67A from *C.lactoaceticus* were obtained and characterized. Xyn10A and Agu67A showed optimum temperature of 80°C and 75°C, respectively. Xyn10A also had good thermostability (75°C and 80°C for 6 h) and broad pH stability (4.5–8.5). Xyn10A could hydrolyze branched xylan and produce xylose, xylobiose, and MeGlcA decorated XOs. Agu67A was active on MeGlcA decorated XOs, and produced MeGlcA and equivalents XOs. The synergistic activity of Xyn10A and Agu67A was detected with both MeGlcA branched xylan and XOs as substrates, and produced xylose, xylobiose, and MeGlcA. The synergistic function of Xyn10A and Agu67A provided a promising way for degrading natural xylan at high temperature. The characterization of the two intracellular GHs also offered an opportunity to systematically evaluate the mechanisms for xylan utilization of *C. lactoaceticus*.

## Materials and Methods

### Strains, plasmids and chemicals

*C. lactoaceticus* DSM 9545 was purchased from DSMZ (Braunschweig, Germany). *E. coli* Top10 (TianGen, China) and plasmid pET-28b (Novagen, USA) were used for gene cloning, and *E. coli* BL21 (DE3) was used for protein expression. Beechwood xylan was purchased from Sigma-Aldrich (St. Luis, USA), and XOs (DP, 2–7) was a kind gift from Longlive Bio-Technology Co. (Shandong, China). D-xylose, locust bean gum (LBG), soluble starch, Avicel, carboxymethyl cellulose (CMC), and chemicals for buffer preparations were obtained from Kepujia Reagent Co. (Beijing, China). All other chemicals were of analytical grade unless otherwise stated.

### Genomic DNA extraction and amplification

The genomic DNA of *C. lactoaceticus* 6A was extracted from 5 mL culture using TIANamp Bacteria DNA Kit (TianGen, China). Based on the whole genome of strain 6A [8], gene *xyn*10A encoding a hypothetical endo-β-1,4-xylanase [GenBank: YP_004798927.1] and gene *agu*67A encoding an α-glucuronidase [GenBank: YP_004798856.1] were predicted and primers were designed as follows: *xyn*10A-F (5′-CTAGCTAGCATGGCTAATTATGAGCATC-3′, *Nhe* I site underlined), *xyn*10A-R (5′-CCCAAGCTTTTAAAGAGTAATTTCAATAAACTTG-3′, *Hind* III site underlined), *agu*67A-F (5′-GCCGCGCGGCAGCATGATTTTATCAAGTAGCAGTAAC-3′), and *agu*67A-R (5′-GCGGCCGCAAGCGTTTATGGATATATCACTCTTC-3′). The PCR mixture contained genomic DNA 1 µL, forward primer 1 µL, reverse primer 1 µL, 2×Pfu PCR MasterMix (TianGen) 12.5 µL, and ddH_2_O 9.5 µL. PCR conditions were as follows: 94°C 5 min, 30 cycles of 94°C 30 s, 55°C 30 s and 72°C 2 min, followed by one cycle of 72°C 5 min. The target PCR products were purified with TIAN gel Midi Purification Kit (TianGen).

### Construction and sequencing of the expression vector

The purified PCR products of *xyn*10A amplification were then digested with *Nhe* I and *Hind* III (Takara, Dalian, China) and inserted into pET-28b at the corresponding sites, obtaining the plasmid pET-28b-*xyn*10A. The purified PCR products of *agu*67A amplification were then digested with T4 DNA polymerase (Takara) and sub-cloned into pET-28b EK/LIC vector, yielding the plasmid pET-28b-*agu*67A. Both two plasmid were transferred into *E.coli* Top10 competent cells by heat shock and grown overnight at 37°C on Lysogeny Broth (LB) agar broth containing kanamycin (50 µg/mL). Positive recombinants were screened by using colony PCR and sequenced with T7 primers from both strands.

### Expression and purification of Xyn10A and Agu67A

The recombinant plasmids were then extracted using TIANprep Mini Plasmid Kit (TianGen) and transformed individually into *E. coli* BL21 (DE3) by heat shock and grown overnight at 37°C on LB agar plates supplemented with kanamycin (50 µg/mL). Seed culture was prepared by growing one single colony separately at 37°C on a rotary shaker (220 rpm) for overnight in 5 mL LB liquid medium containing kanamycin (50 µg/mL). The pre-cultures were then diluted individually 100-fold in fresh LB with antibiotic and cultured at 37°C on a rotary shaker (220 rpm). Isopropyl-β-D-thiogalactopyranoside (IPTG) was added to a final concentration of 0.1 mM when the culture reached an optical density of 0.4-0.6 at 600 nm, and incubations were further continued for an additional 16 h at 16°C. The cells were harvested by centrifugation at 4000 rpm for 15 min at 4°C, and re-suspended in binding buffer (50 mM Tris-HCl pH 7.5, 300 mM NaCl). The cell suspensions were lysed by sonication and centrifugation at 10,000 g for 15 min at 4°C. The supernatants were heated at 65°C for 30 min and centrifuged at 10,000 g for 15 min at 4°C to remove *E. coli* heat labile proteins. The supernatants were loaded individually onto a His-Tag Ni-affinity resin (National Engineering Research Centre for Biotechnology, China) pre-equilibrated with binding buffer for five times. Thereafter, the columns were washed with binding buffer for three times to remove the unbound proteins. Finally, the binded target proteins were eluted from the column with elution buffer (50 mM Tris-HCl pH 7.5, 300 mM NaCl, 150 mM Imidazole). The purity of the target proteins were verified by sodium dodecyl sulfate-polycrymide gel electrophoresis (SDS-PAGE) as described by Laemmli [36]. The native Mws of Xyn10A and Agu67A were analyzed by size exclusion chromatography using a 33 mL elution volume Superdex 200 exclusion column. Gel standard protein mixture, or 300 µL of samples was loaded individually onto the column pre-equilibrated with citrate buffer (50 mM sodium citrate, 150 mM NaCl, pH 6.0) at a flow rate of 0.5 mL/min using Huxi chromatographic separation system (Huxi analysis instrument factory, Co. LTD, Shanghai, China). The apparent Mws of two proteins were calculated from the calibration curve of log (Mw) vs. elution volume.

### Enzyme assay and protein determination

To get initial reaction velocity for activity assay, preliminary experiments were conducted at different conditions. Appropriate amount of Xyn10A or Agu67A was incubated with excess substrates, and the hydrolysis was terminated at different time to get a linear curve of production versus reaction time. Xylanase activity was assayed by incubating 0.78 µg purified recombinant enzyme with beechwood xylan (0.5%, *w/v*) in 100 µL citrate buffer (pH 6.5) at 80°C for 2 min. The amount of reducing sugar generated by Xyn10A was determined by using the *para*-hydroxybenzoic acid hydrazide (PHBAH) method with xylose as a standard [37]. And the activity of Agu67A was assayed by incubating 0.9 µg enzyme with MeGlcA decorated XOs (2.0 mg/mL, final concentration) in 100 µL citrate buffer (pH 6.5) at 75°C for 4 min. The released xylose was measured by high performance liquid chromatography (HPLC) using Hi-Plex Ca column (300×7.7 mm, Agilent Technologies, Tokyo, Japan) with HPLC grade water as mobile phase at 0.6 mL/min, and injection volume of 10 µL. One unit of enzyme activity was defined as the amount of enzyme required to liberate 1 µmol of xylose equivalent per minute under the standard assay conditions. The concentrations of purified proteins were determined by Bradford method using bovine serum albumin (BSA) as a standard. All of the experiments were performed in triplicate.

### Substrate specificity assays of Xyn10A and Agu67A

The substrate specificity of Xyn10A and Agu67A were screened respectively with different polysaccharide substrates including beechwood xylan, XOs, LBG, soluble starch, Avicel, and CMC. All of the tested substrates were at a fixed concentration of 1.0% (*w/v*). Both two enzymes were checked for the ability to hydrolyze various substrates using the Congo red assay [38]. The detection agar plates contained 1.0% (*w/v*) substrate and 0.8% (*w/v*) agar with citrate buffer (pH 6.5). Each of 10 µL enzymes was spotted onto the plates and incubated at 60°C for 12 h. After incubation, all the plates were stained with 0.1% (*w/v*) Congo red for 15 min and washed with 1 M NaCl for 3 times. Meanwhile, substrate specificity of the enzymes was also examined by measuring the produce of reducing sugar from those substrates at 80°C and pH 6.5 for 40 min. All the experiments were performed in triplicate.

### Biochemical characterization of Xyn10A and Agu67A

For determination the temperature optimum of Xyn10A, enzyme activity was measured as described above in a temperature range of 40–95°C at pH 6.0. The pH profile of Xyn10A was determined by using phosphate-citrate buffers (pH 4.0–8.5) at 80°C. The optimal temperature and pH of Agu67A was investigated with MeGlcA decorated XOs as substrates as described above. In optimal temperature assay, the assay was conducted at pH 6.5 with temperature of 40-95°C, while the pH profile (4.0–8.5) was conducted at 75°C. To detect the thermostability of Xyn10A, purified Xyn10A was incubated in phosphate buffer (pH 8.5) at 75, 80 and 85°C, respectively, and residual activity under optimal conditions was compared at the moment of 0.5, 1, 2, 3, 4 and 6 h. The pH stability of Xyn10A was measured by assaying the relative activity under optimal conditions after the enzyme was pre-incubated in different pH buffers ranging from 4.0–8.5 at room temperature for 10 h.

In addition, the effects of various additives on the recombinant xylanase activity were assayed by adding 1 mM and 5 mM of various metal ions (NH_4_^+^, Na^+^, K^+^, Mg^2+^, Fe^2+^, Ca^2+^, Fe^3+^, Zn^2+^, Co^2+^, Mn^2+^, Cu^2+^, Ni^2+^), or 1 mM and 5 mM of chemicals (dithiothreitol, ethylenediamine tetraacetic acid) or 0.1% (*v/v*) and 0.5% (*v/v*) of different solvents (β-mercaptoethanol, Triton X-100, sodium dodecyl sulfate) or 5% (*v/v*) and 10% (*v/v*) organic reagents (glycerol, ethanol, isopropanol, butanol) respectively in the reaction mixture and incubating at 80°C for 5 min. The residual enzyme activity was determined at optimal conditions and the xylanase activity without addition of metal ions or chemical reagents was defined as 100%. All the experiments were performed in triplicate.

### Hydrolysis of beechwood xylan and XOs by Xyn10A and Agu67A

The capacity of Xyn10A and Agu67A to hydrolyze beechwood xylan and XOs was assessed by detecting the hydrolysis products using thin-layer chromatography (TLC), reducing sugar assay, and HPLC analysis. Xyn10A (0.5 µM, final concentration) was incubated with different concentrations (0.1–2.0%, *w/v*) of beechwood xylan under optimal conditions for 4 hours. Besides, Xyn10A (2.0 µM, final concentration), or Agu67A (2.0 µM, final concentration), or a Xyn10A and Agu67A mixture (2.0 µM each, final concentration) was incubated with XOs (2.0%, *w/v*) for 4 hours under optimal conditions. To further evaluate the synergistic activity of Xyn10A and Agu67A, Xyn10A (1.75 µM, final concentration), or Agu67A (0.85 µM, final concentration), or a Xyn10A (1.75 µM, final concentration) and Agu67A (0.85 µM, final concentration) mixture was incubated with beechwood xylan (1.0%, *w/v*) for 0.25, 1, 2, 6 and 12 hours in the same way. Each of the control reaction was performed under the same experimental condition except adding the heat denatured enzyme. After reaction, hydrolysis products were centrifuged at 12,000 g for 10 min. Supernatants were spotted on Silica Gel GF254 TLC plates (Kepujia Reagent Co., Beijing, China) and then developed in 1-butanol/acetic acid/H_2_O (10∶5∶1, *v/v/v*) system for 2 hours after drying [16]. Finally, products were visualized by spraying the dried plates with a mixture of methanolic orcinol (0.05%, *w/v*) and sulfuric acid (5.0%, *v/v*) followed by heating at 75°C for 10 min [16]. Xylose (X_1_), xylobiose (X_2_), xylotriose (X_3_), and xylotetraose (X_4_) were used as standards. At the same time, the hydrolysis products of XOs and beechwood xylan were determined by reducing sugar assay using PHBAH method as mentioned above. And the products from the hydrolysis of XOs and 2 h's hydrolysis of beechwood xylan were also detected by HPLC. All the experiments were performed in triplicate.
